# ﻿*Lepocranus* and *Valalyllum* gen. nov. (Orthoptera, Tetrigidae, Cladonotinae), endangered Malagasy dead-leaf-like grasshoppers

**DOI:** 10.3897/zookeys.1109.85565

**Published:** 2022-07-01

**Authors:** Maks Deranja, Niko Kasalo, Karmela Adžić, Damjan Franjević, Josip Skejo

**Affiliations:** 1 University of Zagreb, Faculty of Science, Department of Biology, Division of Zoology, Evolution Lab, Rooseveltov trg 6, HR-10000 Zagreb, Croatia University of Zagreb Zagreb Croatia

**Keywords:** Gondwana, identification traits, mimicry, new genus, new species, new tribe, phylogenetic position, rainforest, taxonomy, Valalyllini

## Abstract

Only two leaf-like pygmy grasshopper species and specimens are known from Madagascar: the Leatherback Pygmy Grasshopper (*Lepocranusfuscus* Devriese, 1991) —which has a relatively low median carina of the pronotum; and the Malagasy Litterhopper (*Valalyllumfolium***gen. et. sp. nov.**), herein described — which has a high median carina. *Lepocranusfuscus* is known from the rainforests around Tampolo, Manakambahiny, and Mahavelona (Foulpointe). The new taxon, *Valalyllumfolium***gen. et. sp. nov.** is known only from the Belanono forest. Both species inhabit northeastern Madagascar. The new species could be rare or not-easy-to-spot in the rainforest leaf litter, where it most probably lives. A new tribe, Valalyllini**trib. nov.**, is described for the two mentioned genera because its members are different from the Caribbean leaf-like Choriphyllini Cadena-Castañeda & Silva, 2019, from the African leaf-like Xerophyllini Günther, 1979, and from the Asian leaf-like Cladonotini Bolívar, 1887. A tabular key to the tribes of Cladonotinae with leaf-like representatives is provided, together with photographs of type specimens of both species belonging to the newly described tribe. The holotype of the new species belongs to the Muséum national d’Histoire naturelle Orthoptera collection, Paris.

## ﻿Introduction

Leaf mimicry in animals has evolved independently a number of times (Skejo et al. 2019). From the leaf-like satanic leaf-tailed gecko (*Uroplatusphantasticus* Boulenger, 1888) to the Indian oakleaf (*Kallimainachus* Boisduval, 1836), this curious morphology certainly survived as an adaptation for avoiding diurnal predators. Anyone who has visited a tropical rainforest will know how unlikely it is to spot a leaf-like critter in diverse leaf litter. Among the pygmy grasshoppers (Tetrigidae), there are several leaf-like species, mostly belonging to the subfamily Cladonotinae (Tumbrinck 2014). Katydids are well-known for their various leaf-like forms (Nickle and Castner 1995) as well.

**Figure 1.**
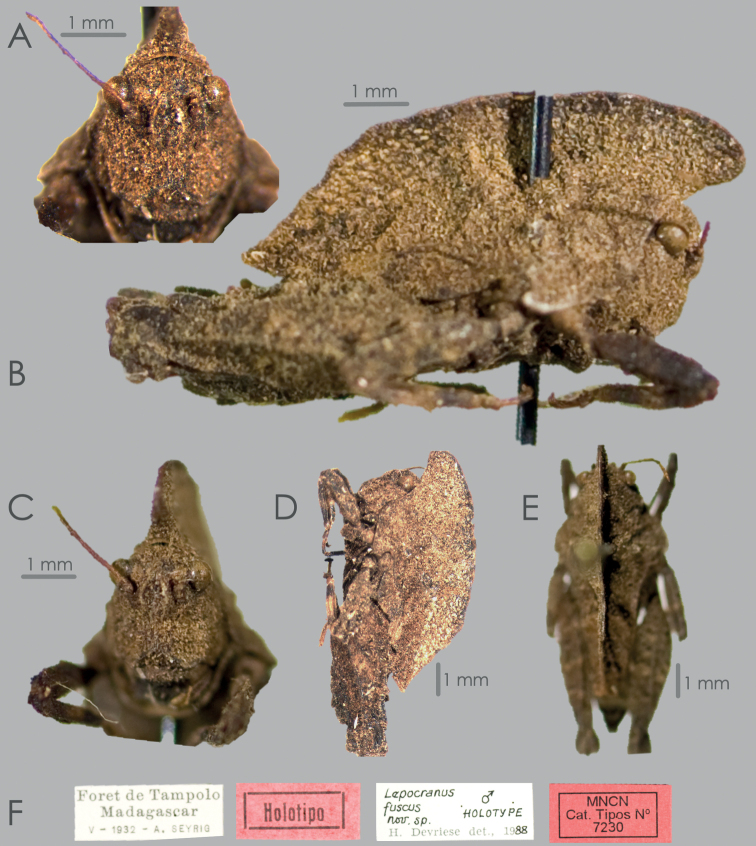
*Lepocranusfuscus* Devriese, 1991 **A** head detail in frontal view **B** habitus in right lateral view **C** habitus in frontal view **D** habitus in light lateral view **E** habitus in dorsal view **F** labels. Photo: J. Skejo and MNCN Madrid.

**Figure 2.**
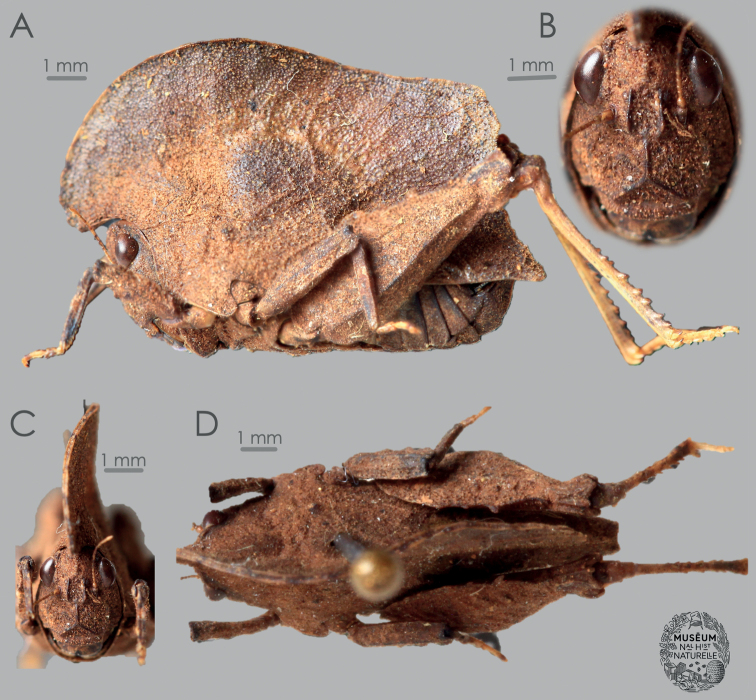
*Valalyllumfolium* gen. et sp. nov. from Madagascar, male holotype, deposited in MNHN Paris. **A** habitus in left lateral view **B** head in frontal view **C** habitus in frontal view **D** habitus in dorsal view. Photo J. Skejo and MNHN Paris.

**Figure 3.**
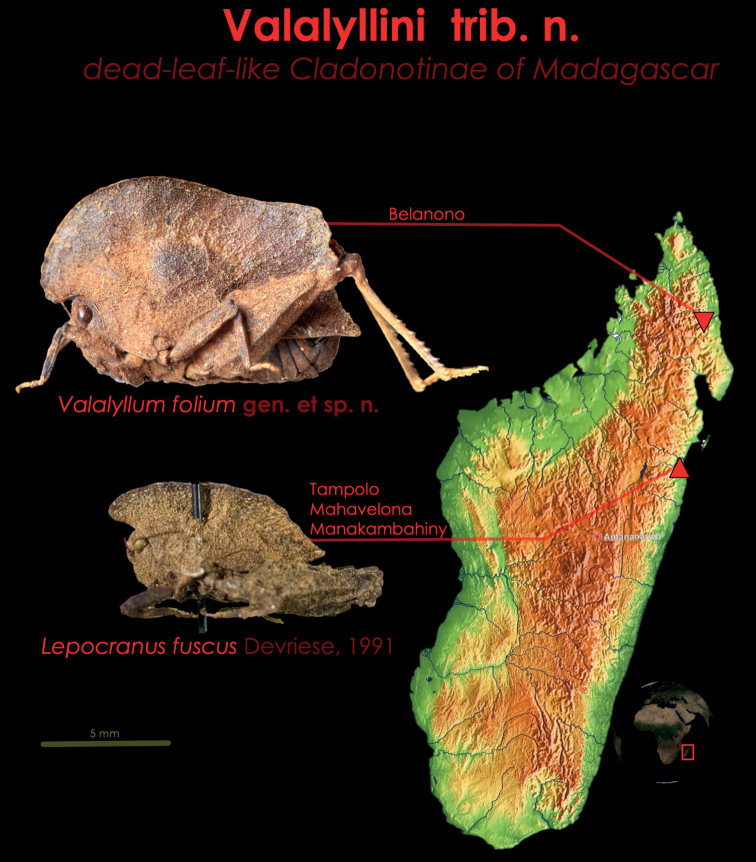
The diversity and the distribution of the tribe Valalyllini trib. nov. (*Valalyllumfolium* gen. et sp. nov. in the north, and *Lepocranusfuscus* in the south), the Malagasy dead-leaf-like Cladonotinae. Both species are endemic to small areas and are likely endangered because of deforestation. Both species most probably inhabit rainforest leaf litter.

Until now, only a single leaf-like species of Tetrigidae was known to inhabit Madagascar—the Leatherback Pygmy Grasshopper, *Lepocranusfuscus* (Devriese 1991) (Fig. 1). This species has been known from several individuals, of which the only digitalized one is a male holotype from the Tampolo forest, slightly northwest of Toamasina (Tamatave). The species has also been reported from the rainforests around Manakamahiny and Mahavelona (Foulpointe) (Danielczak et al. 2017). With this paper, we add one more species to the diverse Malagasy fauna, *Valalyllumfolium* gen. et sp. nov. (Fig. 2). Our description is based on a single male from the Belanono rainforest, in the northernmost rainforests of Madagascar. The aim of this study is to describe *V.folium* sp. nov., compare it to *L.fuscus*, provide identification traits for the Malagasy leaf-like pygmy grasshoppers, and discuss their position in the Tetrigidae tree of life by describing a new tribe, Valalyllini trib. nov. (Fig. 3).

## ﻿Materials and methods

Museum acronyms used were MNCN for Museo Nacional de Ciencias Naturales (Madrid) and MNHN for the Muséum national d’Histoire naturelle. Taxonomy follows the Orthoptera species file (Cigliano et al. 2022) and nomenclature follows the International Code of the Zoological Nomenclature. Morphology and measurements follow Devriese (1991) and Tumbrinck’s (2014) capital work on the subfamily Cladonotinae; we have added a new important character, whether the anterior pronotal tip comes before the face or not. Measurements were taken in ImageJ (version 1.8.0_172) after calibration with millimetre paper. For comparison of the new tribe with already described taxa, we have consulted Devriese (1999) monograph on Xerophyllini Günther, 1979, and the Orthoptera species file (Cigliano et al. 2022) digital catalogue of museum specimens for Choriphyllini Cadena-Castañeda & Silva, 2019 and Cladonotini Bolívar, 1887. The holotype male of *Lepocranusfuscus* is deposited in MNCN and was examined and digitalized by the authors. The holotype male of *Valalyllumfolium* gen. et. sp. nov. is deposited in MNHN, Paris and was examined and digitalized by the authors. Both *Lepocranusfuscus* and *Valalyllumfolium* gen. et. sp. nov. holotypes are now digitalized and high-resolution photographs are uploaded to the Orthoptera species file database (Cigliano et al. 2022).

## ﻿Results

### ﻿Class Insecta

**Order Orthoptera**

**Suborder Caelifera**

**Infraorder Acrididea**

#### Superfamily Tetrigoidea Rambur, 1838

##### ﻿Family Tetrigidae

Taxon classificationAnimaliaOrthopteraTetrigidae

Rambur, 1838

7DC48987-37F5-5925-BBAE-E18188748640

###### Diversity in Madagascar.

Altogether, 76 Tetrigidae species (including the new one) are known to inhabit Madagascar, of which 73 (= 96%) can be found only in Madagascar and nowhere else in the world (Cigliano et al. 2022). These species are assigned to altogether 29 genera, of which 24 are endemic to the island. Hitherto, only a single leaf-like species was known from the island, *Lepocranusfuscus*. This study adds one more species with this interesting cryptic morphology, *Valalyllumfolium* gen. et sp. nov.

##### ﻿Subfamily Cladonotinae

Taxon classificationAnimaliaOrthopteraTetrigidae

BB5D5F6F-6AD2-51C9-898C-7AA5B34501D8

###### Composition and distribution.

This diverse and polyphyletic family includes altogether 314 species assigned to 74 genera (Cigliano et al. 2022). Most genera require revision and are not adequately classified in the Tetrigidae system (Zhang et al. 2020). Within the subfamily Cladonotinae, several monophyletic tribes exist, such as Caribbean Choriphyllini (*Choriphyllum* Serville, 1838 and *Phyllotettix* Hancock, 1902), African Xerophyllini (including leaf-like *Acmophyllum* Karsch, 1890, *Seyidotettix* Rehn, 1938, *Trypophyllum* Karsch, 1890, *Xerophyllum* Fairmaire, 1846), and Asian Cladonotini (*Hymenotes* Westwood, 1837, *Holoarcus* Hancock, 1909, *Dolatettix* Hancock, 1907).

###### Diversity in Madagascar.

Altogether, 18 Tetrigidae species of Madagascar are assigned to the subfamily Cladonotinae: two members of Valalyllini trib. nov. (*L.fuscus* and *V.folium*); 13 species of the genus *Thymochares*, which is currently not assigned to any of the tribes, and it is questionable whether it represents a genus of Cladonotinae; *Microthymocharespullus*, an endemic genus and species with the same taxonomic problem as *Thymochares*; *Epitettixspheniscus* also with the same problematic assignment; and Xerophyllini and its member *Morphopoidesmadagascariensis* (Cigliano et al. 2022).

##### ﻿Tribe Valalyllini

Taxon classificationAnimaliaOrthopteraTetrigidae

trib. nov. [Leaf-like Cladonotinae of Madagascar]

493B2A64-E14F-5A65-8102-7E6E8B131038

http://zoobank.org/49EABF65-9F2F-482B-B545-B1A997AE9957

Figs 1 , 2 , 3 ; Table 1

Cladonotini (partim): Tumbrinck et al. (2020): 336 (tentatively assigned to Cladonotinae: Cladonotini).

###### Type genus.

*Valalyllum* gen. nov., herein described (see below), type species *V.folium* sp. nov., also described herein.

###### Composition and distribution.

Two monotypic genera, *Lepocranus* (including only *L.fuscus*) and *Valalyllum* gen. nov. (including *V.folium* sp. nov.). *Lepocranusfuscus* is endemic to the rainforests east of Mahavelona (Foulpointe) (Danielczak et al. 2017), while *V.folium* sp. nov. is endemic to northern Malagasy rainforests around Sambava and Andapa. *Lepocranusfolium* sp. nov. lives 350 km more northerly than *L.fuscus*.

###### Descriptive diagnosis.

Members of Valalyllini (*Lepocranus*, *Valalyllum* gen. nov.) are very similar to and thus most likely closely related to Asian Cladonotini (genera *Misythus* Stål, 1877, *Hymenotes* Westwood, 1837, *Holoarcus* Hancock, 1909) and Caribbean Choriphyllini (*Choriphyllum* Serville, 1838 and *Phyllotettix* Hancock, 1902). Head, pronotum and legs in these three tribes show remarkable similarity and are regarded as homologous. Superficially, the leaf-like morphology of Valalyllini trib. nov. also resembles that of leaf-like Xerophyllini, but detailed comparisons reveal no homologous parts.

Antenna shorter than hind femur; vertex very wide; vertex slightly and obliquely elevated above the compound eyes; vertex without horns; upper margin of the antennal grove in the level of the lower margin of a compound eye; pronotal tip of the pronotum projected above the head, but not before the eyes or before the face; transverse pronotal veins weak, almost unrecognizable; pronotal tip obliquely bilobate in dorsal view; legs smooth, only with weak (small) undulations and triangular projections, not toothed or sawed. Found only in Madagascar.

Table 1 shows a comparison between leaf-like Caribbean Choriphyllini (*Choriphyllum* Serville, 1838 and *Phyllotettix* Hancock, 1902), Asian Cladonotini (*Hymenotes* Westwood, 1837, *Misythus* Stål, 1877, *Holoarcus* Hancock, 1909 and *Dolatettix* Hancock, 1907), and African Xerophyllini (*Xerophyllum* Fairmaire, 1846, *Trypophyllum* Karsch, 1890, *Acmophyllum* Karsch, 1890). The leaf-like Tetrigidae were visually compared by Skejo et al. (2019). For comparison and elucidation of the mentioned characters, the reader is urged to refer to the abovementioned publication.

**Table 1.** Tabular key to the four Cladonotinae tribes with leaf-like representatives (Valalyllini trib. nov. from Madagascar, Choriphyllini from the Caribbean, Cladonotini from SE Asia, and Xerophyllini from Africa).

	Valalyllini trib. nov.	Choriphyllini	Cladonotini	Xerophyllini
**Distribution**	**Madagascar**	**Caribbean**	**Philippines, Papua**	**Tropical Africa**
**Antenna**	short	long or short	short	short
**Vertex horns**	absent	absent	absent	present
**Upper margin of the antennal groove**	on the level of the lower margin of a compound eye	on the level of the lower margin of a compound eye	on the level of the lower margin of a compound eye	below the lower margin of a compound eye
**Anterior tip of the pronotum**	not falling in front of the face	strongly projected in front of the face	projected in front of the eyes	variable, not falling in front of the face (*Trypophyllum*) or projected in front of the face (*Xerophyllum*)
**Transverse veins on the pronotum**	weak	strong	very strong	weak
**Posterior tip of the pronotum**	obliquely bilobate	pointed	variable (*pointed* in *Misythus*, bilobate in *Cladonotus*)	pointed
**Legs texture**	smooth, with small protrusions	smooth	toothed or spiky	toothed or spiky

##### ﻿Genus Lepocranus

Taxon classificationAnimaliaOrthopteraTetrigidae

Devriese, 1991

23279F64-FB99-5868-B515-7C4CBCCB0D7F

Lepocranus : Devriese 1991

###### Type species.

*Lepocranusfuscus*, by original designation and by the original monotypy.

###### Composition and distribution.

A single species only (*L.fuscus*), endemic to the Malagasy rainforests east of Mahavelona (Foulpointe), such as Tampolo, where the holotype is from, and Manakambahiny (reported in Danielczak et al. 2017).

###### Original etymology.

Complex noun, male in gender, composed of Latinized Ancient Greek “lepos” (λέπος), meaning *bark*, and “chranos” (χράνος), meaning *helmet* (Devriese 1991).

###### Diagnosis.

Same as for the species, see below.

##### ﻿ Lepocranus fuscus

Taxon classificationAnimaliaOrthopteraTetrigidae

Devriese, 1991

8095758E-779D-5E0A-A677-D8E9FFE2671E

Fig. 1

Lepocranus fuscus : Devriese 1991

###### Material examined.

***Holotype*.** Madagascar • 1​​♂; “Foret de Tampolo, Madagascar”; May. 1932; A. Seyrig leg.; MNCN 7230.

###### Genus and species diagnosis.

Small (< 10 mm long), apterous, leaf-like, cryptic species endemic to northern Madagascar. Antennae are short and filiform, composed of 15 antennomeres. The upper margins of the antennal groove are at the lower margin of a compound eye. Frontal costa bifurcates between the eyes into rounded facial carinae (parallel in *Valalyllum* gen. nov.), between which there is a wide scutellum, as wide as a compound eye. Vertex obliquely projected above the eyes in frontal view. Vertex about 3 times wider than a compound eye. In the frontal view, a compound eye is rounded (ovoid in *Valalyllum* gen. nov.). Pronotum is leaf-like, 2.4 times as long as high (1.75 times in *Valalyllum* gen. nov.) because of the compressed and elevated median carina. In the dorsal view, the median carina of the pronotum is sulcate. Median carina of the pronotum is straight in the dorsal view (undulated in *Valalyllum* gen. nov.). Pronotum dips above the head in dorsal view, then smoothly curves upwards and gradually descends towards the posterior apex at an obtuse angle (150° slope, already reported in Devriese 1991) (posterior slope is abrupt in *Valalyllum* gen. nov. and forms a right-angle). Posterior slope of the median carina is weakly undulated (much more in *Valalyllum* gen. nov.). The posterior apex of the pronotum is bilobated in the dorsal view. Pronotum does not cover the whole abdomen, the last segments are not covered, and the subgenital plate is visible (fully covered in *Valalyllum* gen. nov.). Dorsal margin of hind femora bearing strong projections (lappets). Genicular and antegenicular teeth are large and blunt (small and angular in *Valalyllum* gen. n). Mid tibia is stout. The top margins of the mid and the fore femora undulated but lack strong tubercules. Pulvilli of the hind tarsi are rounded.

###### Original etymology.

Latin adjective in male gender, “fuscus, -a, -um” meaning brown (Devriese 1991).

###### Vernacular name.

Leatherback Pygmy Grasshopper (Danielczak et al. 2017).

###### Measurements

**(male holotype). *Body length***: (from the tip of the head to the tip of the subgenital plate) 8.6 mm (cited 9.8 mm in Devriese 1999, from the tip of the pronotum to the tip of the subgenital plate). ***Pronotum length***: 8.1 mm (cited 8.2 mm in Devriese 1991). ***Pronotum maximum height***: 3.3 mm. ***Pronotum width between the lateral lobes***: 3.8 mm. ***Pronotum width between the shoulders***: 2.5 mm. ***Eye width***: 0.4 mm. ***Vertex width***: 1.2 mm. ***Fore femur length***: 2.2 mm. ***Fore femur width***: 0.5 mm. ***Mid femur length***: 2.5 mm. ***Mid femur width***: 0.8 mm. ***Hind femur length***: 5.3 mm (cited 5.3 mm in Devriese 1991). ***Hind femur width***: 2.1 mm. ***Hind femur length/width ratio***: 2.5.

###### Locus typicus.

Madagascar, Tampolo.

###### IUCN Red List Assessment.

The Leatherback Pygmy Grasshopper was listed as an endangered species on the IUCN Red List because (1) the minimal geographic range it inhabits (the extent of occurrence is only about 3000 km^2^), (2) the population seems to be fragmented, and (3) the decline in both the number of mature individuals and the size and quality of the range area due to inferred severe deforestation (Danielczak et al. 2017).

##### ﻿Genus Valalyllum gen. nov.

Taxon classificationAnimaliaOrthopteraTetrigidae

87DE89AA-490B-5D4C-8F23-FFC7987FA32C

http://zoobank.org/40A42840-9E96-4ED6-8D4A-2872D80E2E70

###### Type species.

*Valalyllumfolium* gen. et sp. nov., here described, by original monotypy.

###### Composition and distribution.

A single species only (*V.folium*), endemic to Madagascar (Belanono).

###### Etymology.

Noun of neuter gender. From Malagasy “*valala*” or “*vahalala*”- grasshopper and Latinized Ancient Greek “*phyllum*” - leaf

###### Diagnosis and description.

Same as for the species, see below.

##### ﻿ Valalyllum folium sp. nov.

Taxon classificationAnimaliaOrthopteraTetrigidae

45EFED13-0339-508A-A55A-D86E2BB1A48E

http://zoobank.org/0DA809A6-3F8A-48D2-8DAB-2E135114FC73

Fig. 2

###### Material examined.

***Holotype*.** Madagascar • 1 ♂; East Madagascar, Belanono, “30 km SW de Sambava, sur la route d’Andapa” [along the road to Andapa]; Valdon and Peyrieras leg.; MNHN.

###### Diagnosis.

Large (> 11 mm long), apterous, leaf-like, rectangular, and cryptic species endemic to northern Madagascar. Antennae are short and filiform, composed of 15 antennomeres. Upper margins of the antennal groove in the level of the lower margin of a compound eye. Frontal costa bifurcates between the eyes into parallel facial carinae (more rounded in *Lepocranus*), between which there is a wide scutellum, as wide as a compound eye. Vertex obliquely projected above the eyes in the frontal view. Vertex about 3 times wider than a compound eye (measured at its widest part as seen in the frontal view). In the frontal view, a compound eye is ovoid (rounded in *Lepocranus*). Pronotum is rectangular and 1.75 times as long as high (2.4 times in *Lepocranus*) because of the compressed and elevated median carina. In the dorsal view, the median carina of the pronotum is sulcate. Median carina of the pronotum is undulated in a sinusoid fashion in the dorsal view (straight in *Lepocraus*). Pronotum dips above the head in dorsal view, then smoothly curves upwards and sharply descends towards the posterior apex. The posterior slope of the pronotum is abrupt (gradual in *Lepocranus*), forming a right-angle (obtuse angle in *Lepocranus*), and undulated. The posterior apex of the pronotum is bilobated in the dorsal view. Pronotum covers the whole abdomen (last segments not covered in *Lepocranus*). Dorsal margin of the hind femora bears three small projections (lappets). Genicular and antegenicular teeth are small and angular. Mid tibia is stout. Top margins of the mid and the fore femora lack tubercules. Pulvilli of the hind tarsi are rounded.

###### Etymology.

The specific epitheton is a noun in apposition, from Latin “*folium, -i, n.*” leaf, because of the species’ leaf-like morphology.

###### Proposed vernacular name.

Malagasy Litterhopper

###### Description.

***Holotype* (male). *General appearance*.***Valalyllumfolium* gen. et sp. nov. is a large (> 11 mm); smooth; rectangular; cryptic; dead-leaf-mimicking species with fine leaf-like venation on the elevated part of pronotum; uniformly brown except for the yellowish tarsi of all legs, as well as pale-yellowish hind tibiae.

***Head*.***Antenna* (Fig. 2B, C) short, filiform, composed of 15 antennomeres. The first segment is the largest scapus, second is a barrel-like pedicel, both circular in cross-section, while the remaining 13 antennomeres make up the flagellum. Segments 3^rd^ to 6^th^ are basal segments of the flagellum and they are robust, about two times as long as wide. Segments 7^th^ to 10^th^ are the central segments, and they are elongated, from four to five times as long as wide. Segments 11^th^ to 13^th^ are the subapical segments, shorter and bulkier than the mid segments. Last two antennomeres, i.e., 14^th^ and 15^th^ are reduced apical antennomeres. In the frontal view (Fig. 2B, C), the vertex is 3 times wider than the width of the compound eye (1.2 mm wide vertex, 0.4 mm wide compound eye); the vertex tip is above the top margin of the compound eyes, forming a smooth convex bulge. Frontal costa is smooth before the bifurcation, without visible projections or teeth. Bifurcation of the frontal costa is situated just below the line connecting the mid portion of the compound eyes. Height of the compound eye is greater than the height of the scutellum. Compound eye is ovoid. Width of the compound eye is the same as the width of the scutellum. Scutellum in its widest part and at the level of antennal groove is significantly wider than the antennal groove. Facial carinae are visible, and run straight after the bifurcation. Facial carinae are however, slightly sub-parallel, so the scutellum is slightly widened at the level of the median ocellus. Facial carinae are compressed and elevated but smooth. Paired ocelli are situated below the line connecting the mid portion of the compound eyes, but above the lower margin of compound eyes. Top margin of the antennal groove is placed at the level of the bottom margin of the compound eyes. In the lateral view (Fig. 2A), both vertex and face are visible around an ovoid compound eye. Fastigium protrudes beyond the furthest margin of the compound eyes for a half of the compound eye width. Occipital area is visible and narrow. In the dorsal view (Fig. 2D), the vertex is 3 times wider than the width of the compound eye. However, in this view, the vertex is also visibly very short and reaches to about a third of the compound eye length, i.e., it is not projected, but indrawn. Lateral and transverse carinae are slightly visible and elevated. Medial carina not visible due to pronotal occlusion.

***Pronotum*** In the frontal view (Fig. 2C), the pronotum is visible above the head as a large, compressed and elevated and undulated projection with sulcated (ditched) ridge. In lateral view (Fig. 2A), the pronotum has the appearance of a dead leaf, because of the strongly compressed and elevated median carina of the pronotum. Prozonal and extralateral carinae are invisible. The pronotum is long (13.4 mm) and high (7.6 mm), giving a rectangular shape to the insect. Tip of the median carina of the pronotum has striped pale-dark-pale-dark coloration. Anterior margin is projected above the head as an oblique projection and then goes in circular fashion towards the dorsal portion, where it becomes the highest in the level of the mid coxae. After its highest portion, the dorsal margin of the median carina slowly decreases in height, and then in the level of the subgenital plate it abruptly falls in almost rectangular angle. The whole distal portion of the pronotum after this abrupt slope is finely toothed and undulated. Ventral sinus of the pronotum is large and visible, while tegminal sinus lacks because of the absence of wings. Infrascapular area, covered by the hind femora, is wide, smooth and convex. The compressed dorsum of pronotum is finely granulated and intercepted with many fine veins (carinulae), giving an insect even more credible dead-leaf mimicry. Paranota are triangular, with truncate-oblique apex. In the dorsal view (Fig. 2D), the median carina has a sulcated ridge and forms a sinusoid undulation from the head caudad. The pronotum is finely granulated. Prozonal and extralateral carinae are absent, not visible. No pronotal projections visible. Shoulders (humeral angles) oblique, not projected outwards. Posterior apex of the pronotum is widely bilobate.

***Legs*. *Fore legs*** (Fig. 2A, C, D). The fore femur is stouter than the mid one, length/width ratio is about 3.1. The dorsal margin of the fore femur has a continuous carina without tubercles. Ventral margin with the same femur has one tubercle close to the femur’s distal end. The fore tibia is finely serrated and rectangular in cross-section. Distal segment of the fore tarsus is much longer than the proximal one. ***Mid legs*** (Fig. 2A, D). Dorsal and ventral margins of the mid femur bear continuous carinae, but ventral carina also has three small tubercles. Length/width ratio of the mid femur is 3.7. The mid tibia is finely tuberculated and rectangular in cross-section. Distal segment of the fore tarsus is much longer than the proximal one. ***Hind legs*** (Fig. 2A, D). The dorsal margin of the hind femur bears three large, but relatively blunt teeth. Ventral margin with continuous carina. Length/width ratio of the hind femur is 2.7. The hind tibia bears numerous strong teeth, but otherwise has a very smooth surface. First segment of the tarsus bears three strongly protruding, smooth and rounded pulvilli. First tarsal segment is much longer than the third.

###### Measurements

**(male holotype). *Body length*** (from the tip of head to the tip of the subgenital plate) 11.3 mm. ***Pronotum length*** 13.4 mm. ***Pronotum maximum height*** 7.6 mm. ***Pronotum width between lateral lobes*** 5.2 mm. ***Pronotum width between the shoulders*** 3.5 mm. ***Eye width*** 0.4 mm. ***Vertex width*** 1.2 mm. ***Fore femur length*** 2.5 mm. ***Fore femur width*** 0.8 mm. ***Mid femur length*** 3.3 mm. ***Mid femur width*** 0.9 mm. ***Hind femur length*** 7.5 mm. ***Hind femur width*** 2.8 mm. ***Hind femur length/width ratio*** 2.7.

###### Locus typicus.

Madagascar, Sava region, Belanono (rainforest between Sambava and Adapa).

###### Proposed IUCN Red List Assessment.

Similar to the Leatherback Pygmy Grasshopper (*Lepocranusfuscus*) (Danielczak et al. 2017), the Malagasy Litterhopper (*Valalyllumfolium* gen. et sp. nov.) should be immediately listed as an endangered species in the IUCN Red List because (1) of a very small geographic range it inhabits (known from a single locality, (2) the population might be fragmented and (3) the decline in both the number of mature individuals and the size and quality of the range because of expected severe deforestation.

## ﻿Discussion

Despite its relatively small area, Madagascar is one of the richest biodiversity hotspots on Earth, boasting a high number of endemic taxa from the species level and above. This fact characterizes Madagascar as a high-priority area for conservation (Myers et al. 2000). Even at the level of Tetrigidae we find unbelievable diversity, with the new genus being the 24^th^ endemic genus of the total number of 29 (Cigliano et al. 2022). Tragically, Madagascar has a long tradition of deforestation. Only 15% of the island remains forested, with the northern rainforest being the healthiest (Ganzhorn et al. 2001; Harper et al. 2007; Vieilledent et al. 2018). The deforestation continues despite efforts to stop it (Tabor et al. 2017). Undescribed species can hardly be protected (Liu et al. 2022) and our models hinge on abundant and reliable data (Tedesco et al. 2014; Régnier et al. 2015). The nomenclatural acts we perform reflect our endeavor to assist in preservation of biodiversity.

The two members of the Valalyllini trib. nov. are similar but can be readily separated by several characters, namely the size and the shape of the eyes, the size of the leaf-like crest, and the general size of the body. Although these features can be variable individually, *L.fuscus* as a whole appears as a neotenic form of *V.folium* gen. et sp. nov. and is thus likely closely related to it but morphologically consistently separate. A remarkable similarity between the leaf-like tribes Valalyllini trib. nov. (Madagascar), Cladonotini (Asia), and Choriophyllini (Caribbean) is evident, so it is reasonable to hypothesize that those characters represent homologies and reflect the common ancestry on the ancient continent of Gondwana, which is a pattern that has been observed in numerous other taxa (Turner 2004; Gray et al. 2009; Reguero and Goin 2021). Cladonotinae are polyphyletic; their taxonomy is still far from resolved (Zhang et al. 2020). There is a strong possibility that more tribes could be defined within the subfamily and that some of the defined ones could contain genera that better fit elsewhere. The broad picture we present could lead to the solution, but there is more work to be done.

Both *Lepocranusfuscus* and *Valalyllumfolium* gen. et sp. nov. are known only from a small area. A single digitalized specimen of *L.fuscus* is not exceptionally well preserved due to the presence of mould (Devriese 1991) and the new species is known only from a single specimen. Clearly, this situation is not ideal as the identity of both species remains incomplete. *Lepocranusfuscus* is already considered endangered (Danielczak et al. 2017), and could become extinct before we know anything about it. The same could be true for *V.folium* sp. nov. Considering the previously discussed points, a clear distinction between two Malagasy dead-leaf-mimic genera is presented. It is certainly going to be updated in the light of new findings as it is definitely incomplete, but it serves as a valuable starting point for future research.

## ﻿Conclusions

A new dead-leaf-like genus and species of Malagasy Tetrigidae, *Valalyllumfolium* gen. et sp. nov. is described and compared to *Lepocranusfuscus*, the only species of the genus *Lepocranus*. The two species are similar, but clearly separable by the general size, the shape of the pronotal crest and the shape of the eyes. *Valalyllumfolium* gen. et sp. nov. and *Lepocranusfuscus* are assigned to the newly described tribe Valalyllini trib. nov. as the only Malagasy tetrigids with leaf-like pronotal crests. The comparison of the present tribe with others of similar morphology has revealed several likely homologies, which imply common ancestry. As the taxonomy of Cladonotinae is not resolved yet, new groups within the subfamily are certainly going to be defined, which will help in elucidating their evolutionary relationships.

*Lepocranusfuscus* is already considered endangered according to the IUCN Red List and, following the same pattern, we propose that the new species should be considered endangered as well.

Both species from the newly described tribe are defined only by a single specimen each, which is a fact that obscures the variability of the species. However, considering the rampant deforestation of Madagascar and the fact that the species can be differentiated by intraspecifically invariable characters homologous with other leaf-like tribes, we find it vital to describe the new, likely endangered, species and thus assist the efforts to classify and protect the rich Malagasy biodiversity.

## Supplementary Material

XML Treatment for Tetrigidae

XML Treatment for Cladonotinae

XML Treatment for Valalyllini

XML Treatment for Lepocranus

XML Treatment for Lepocranus fuscus

XML Treatment for Valalyllum

XML Treatment for Valalyllum folium
